# Changes in the microbial community of semen exposed to different simulated forensic situations

**DOI:** 10.1128/spectrum.00125-24

**Published:** 2024-07-09

**Authors:** Yangyang Zheng, Linying Ye, Jieyu Du, Litao Huang, Miaoqiang Lun, Meiyun He, Guichao Xiao, Weian Du, Chao Liu, Ling Chen

**Affiliations:** 1 Guangzhou Key Laboratory of Forensic Multi-Omics for Precision Identification, School of Forensic Medicine, Southern Medical University, Guangzhou, Guangdong, China; 2 Guangdong Homy Genetics Incorporation, Foshan, China; 3 National Anti-Drug Laboratory Guangdong Regional Center, Guangzhou, China; Dominican University New York, Orangeburg, New York, USA

**Keywords:** body fluid identification, forensic medicine, microbiome, semen exposure, 16S rRNA gene sequencing

## Abstract

Semen is one of the common body fluids in sexual crime cases. The current methods of semen identification have certain limitations, so it is necessary to search for other methods. In addition, there are few reports of microbiome changes in body fluids under simulated crime scenes. It is essential to further reveal the changes in semen microbiomes after exposure to various simulated crime scenes. Semen samples from eight volunteers were exposed in closed plastic bags, soil, indoor, cotton, polyester, and wool fabrics. A total of 68 samples (before and after exposure) were collected, detected by 16S rDNA sequencing, and analyzed for the microbiome signature. Finally, a random forest model was constructed for body fluid identification. After exposure, the relative abundance of *Pseudomonas* and *Rhodococcus* changed dramatically in almost all groups. In addition, the treatment with the closed plastic bags or soil groups had a greater impact on the semen microbiome. According to the Shannon indices, the alpha diversity of the closed plastic bags and soil groups was much lower than that of the other groups. Attention should be given to the above two scenes in practical work of forensic medicine. In this study, the accuracy of semen recognition was 100%. The exposed semen can still be correctly identified as semen based on its microbiota characteristics. In summary, semen microbiomes exposed to simulated crime scenes still have good application potential for body fluid identification.

**IMPORTANCE:**

In this study, the microbiome changes of semen exposed to different environments were observed, and the exposed semen microbiome still has a good application potential in body fluid identification.

## INTRODUCTION

Sexual assault cases account for a high proportion of criminal cases, in which semen is a common biological sample. Therefore, identifying body fluids from suspicious stains at crime scenes is conducive to reconstructing crime scenes and determining the nature of a case, which is highly significance for detecting sexual crimes.

At present, microscopic examination and prostate-specific antigen (PSA) test paper are commonly used methods for semen examination in forensic practice. The former is regarded as the gold standard for semen confirmation, but it has certain limitations for azoospermia (1). The PSA strip method is simple and fast, which is usually used for the initial screening of suspicious samples. It is important to note that PSA is not a confirmatory test (2). To explore new markers for semen identification, researchers have developed molecular genetics-based methods using gene-targeted mRNA, microRNA, or DNA methylation (3, 4). However, the stability of these methods is easily affected by various factors, such as the environment, age, disease, and lifestyle (5).

In addition, microorganisms show certain application potential in the traceability of tissue and body fluids in forensic medicine (6). At present, the samples of body fluid identification research based on the characteristics of the human microbial community composition are based mainly on fresh body fluid (7), and few studies have been conducted on tissue or body fluid exposure. In practice, body fluids are usually exposed to the environment for a period of time before extraction. Dobay et al. (8) and our previous studies (9) showed that the microbial characteristics of semen after exposure were relatively stable. The design of the above experiment was relatively simple, as described below, and swabs were only placed on the test tube rack and exposed to the indoor environment for a certain period of time. Semen is usually left in bed sheets, clothes, or plastic bags and is exposed to the environment of the crime scene. Therefore, in order to be more similar to the actual case, it is necessary to further improve the experimental design on the basis of previous experiments, simulate a variety of exposure environments that may occur in actual cases, and explore the impact of additional exposure factors.

In this study, semen samples from eight healthy individuals were collected and exposed for 15 days in plastic bags, indoor, soil, cotton carrier, wool carrier, and polyester fiber carrier environments. Using 16S rRNA gene sequencing, we aimed to explore the changes in semen microorganisms after exposure to different environments.

## MATERIALS AND METHODS

### Sample collection

**Fig 1:**
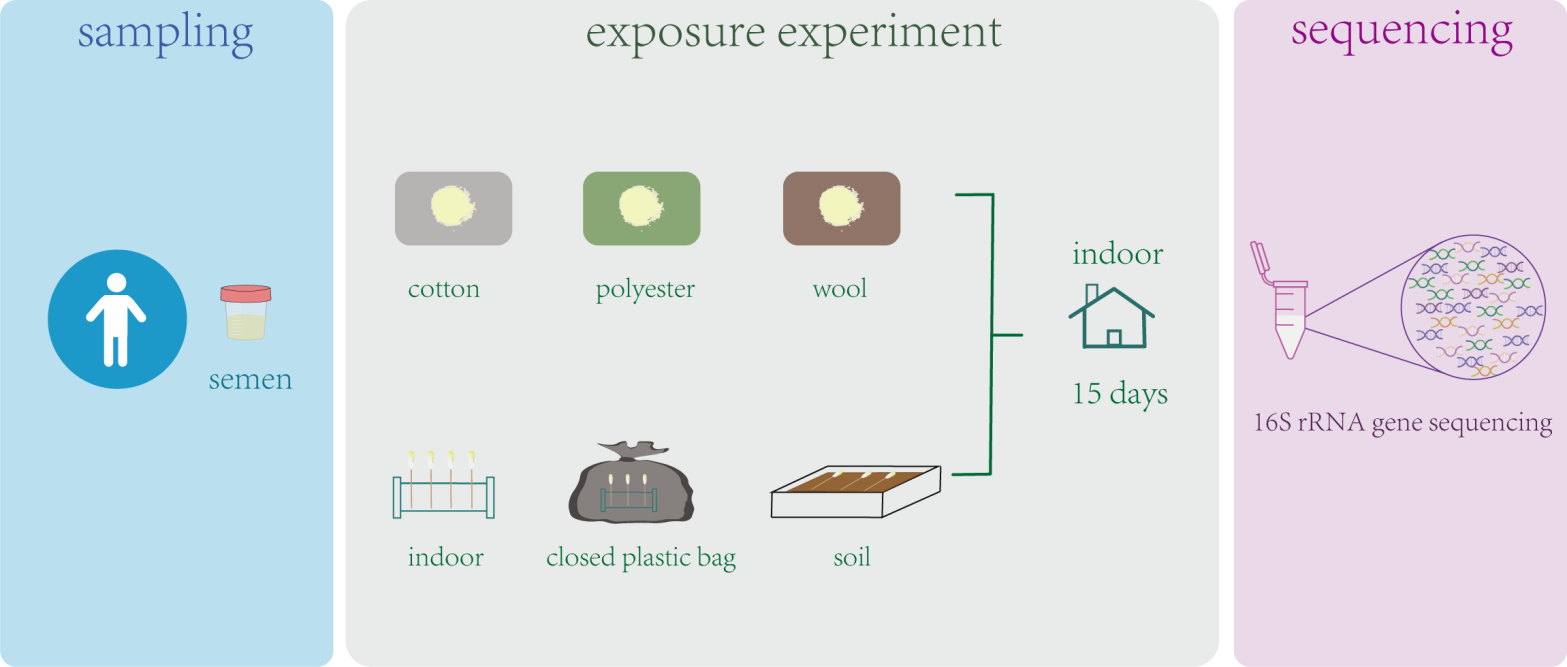
Experimental design. Semen collected from the same volunteer was dropped on the carriers of different materials (cotton, polyester, and wool fabric) or on the sterile cotton swabs placed on soil, closed plastic bags, and indoor environments. Then 16S rRNA gene sequencing was performed after 15 days of exposure.

This study was approved by the Biomedical Ethics Committee of Southern Medical University, Guangzhou, China. After obtaining informed consent, we collected semen samples from eight individuals. All volunteers had no obvious urogenital complications or symptoms of sexually transmitted diseases and had no antibiotics for a month. Strictly disinfect the hands and glans with alcohol before sampling. After 3 days of abstinence (masturbation and sex were all prohibited), semen was collected by masturbation. The semen collected from the same volunteer was divided into seven portions, each containing 0.5 mL of semen, and divided into groups, such as (10) positive control group: one part of the semen was placed in sterile test tube and immediately stored at −80℃ (5); treatment group: semen was dropped on sterilized carriers of different materials (cotton, polyester, and wool fabric), or semen was dropped on sterile cotton swabs and placed on soil, closed plastic bags (CPB), and indoor environment. Then they were exposed for 15 days (Fig. 1). In addition, the negative control involved sterilized carriers and sterile cotton swabs exposed to the same environmental conditions as the treatment groups, whereas the blank control consisted of sterilized carriers and swabs that were not subjected to any exposure experiments. The ambient temperature to which the sample was exposed ranged from 20℃ to 25℃.

### DNA extraction, PCR amplification, and sequencing

According to the manufacturer’s instructions, the E.Z.N.A. Soil Omega Kit (Omega Bio-Tek, Norcross, Georgia, USA) was used to extract total DNA. The quality of DNA extraction was checked by 1% agarose gel electrophoresis and stored at −20°C. A Qubit dsDNA HS Assay Kit was used to detect the DNA concentration on a Qubit 2.0 fluorometer. For all qualified DNA samples, primers 338F (5′-GGACTACHVGGGTWTCTA AT-3′) and 806R (5′-GGACTACHVGGGTWTCTAAT-3′) were used to amplify the V3-V4 region of the 16S rRNA gene. Each PCR (20 µL) contained 4 µL of 5× FastPfu buffer, 2 µL of 2.5 mM dNTPs, 0.8 µL of each primer (5 µM), 0.4 µL of FastPfu polymerase, and 10 ng of template DNA. The PCR cycle conditions were an initial step of 95°C for 3 minutes, followed by 27 cycles of 95°C (30 s), 55°C (3 s), and 72°C (30 s). After the cycle was completed, the reaction was incubated at 72°C for 10 minutes. A thermal cycling PCR system (GeneAmp 9700, ABI, USA) was used for the amplification. After PCR was completed, the amplicon concentration was normalized and combined using a SequalPrep DNA Normalization Plate (Invitrogen, Maryland, USA). The NEXTFLEX Rapid DNA-Seq Kit was used to construct a library of purified PCR products. After library quality control and quantification, sequencing was performed using MiSeq kit V3 (Illumina, San Diego, California, USA) on the Illumina MiSeq PE300 platform. The raw data were uploaded to the NCBI SRA database (accession number: PRJNA1102678).

### Bioinformatics analysis

FLASH software (version 1.2.11) was used for DNA sequence assembly; QIIME (version 1.9.1) was used to generate each taxonomic abundance table and then calculate the beta diversity distance. Through the software platform, UPARSE was made (version 7.1) (http://drive5.com/uparse/) to perform OTU clustering. With a similarity above 97%, an OTU table was generated. The RDP classifier (version 2.11) (https://sourceforge.net/projects/rdp-classifier/) and the SILVA database (version 138) were used for classification. Mothur software (version 1.30.2) was used to calculate the alpha diversity and evenness index of the microbial community. R software (version 3.3.1) was used to generate various diagrams, such as rarefaction curves, Venn, arplot, and PCoA. The stats package in R language (version 3.3.1) and the scipy package in Python were used for the Wilcoxon rank sum test. It can determine the significant differences of species with different sample groups of semen and correct the *P* value by various methods. LEfSe(R language version 3.3.1) was used to perform linear discriminant analysis (LDA) aiming to analyze the significant differences among the different treatments of semen groups.b

### Machine learning process

R software (version 3.3.1) was used to construct a random forest (RF) model based on the species and the relative abundance of microorganisms at the genus level. Fifty-six samples with semen were included in this study, including a positive group and treatment group. Other samples, such as 27 skin samples, 30 saliva samples, and 66 vaginal secretions, were obtained from our previously published articles (11, 12). The model included a total of 179 samples, of which 70% (126) were randomly selected as the training set, and 30% (53) were selected as the testing set. Five hundred decision trees were set to construct the model. The mean decrease accuracy was used to evaluate the importance of the genus as an indicator. The higher the value, the more important the genus was in the classification model. The validation method used in this model was 10-fold cross-validation. The number of important features required for this model was determined when the prediction error rate was lowest.

## RESULTS

### Summary of semen samples

Semen samples were collected from eight unrelated male individuals, and the groups were as follows: positive control (8 samples), 6 treatment groups (48 samples), negative control (6 samples), and blank control groups (6 samples). PCR amplification and high-throughput sequencing were performed on the V3-V4 regions of the 16S rRNA genes of the above 68 samples. The blank control did not show any amplification products using gel electrophoresis, and further sequencing of the PCR products also did not yield results. PCR amplification and sequencing success were 100% for positive control, treatment group, and negative control group. Therefore, the total number of valid sequences obtained from 62 samples is 2,507,723. By clustering the sequences at the 97% similarity level, 5,569 original data OTUs were classified into 1 domain, 1 kingdom, 45 phyla, 131 classes, 315 orders, 547 families, 1,247 genera, and 2,232 species (Table S1). The Good’s coverage for the observed OTUs was 0.98 ± 0.02 (mean ± sd), and the rarefaction curves showed a tendency toward saturation with increasing number of reads, indicating that the amount of sequencing data was large enough to reflect the vast majority of microbial species information in the samples (Fig. S1).

### Alpha diversity analysis

**Fig 2:**
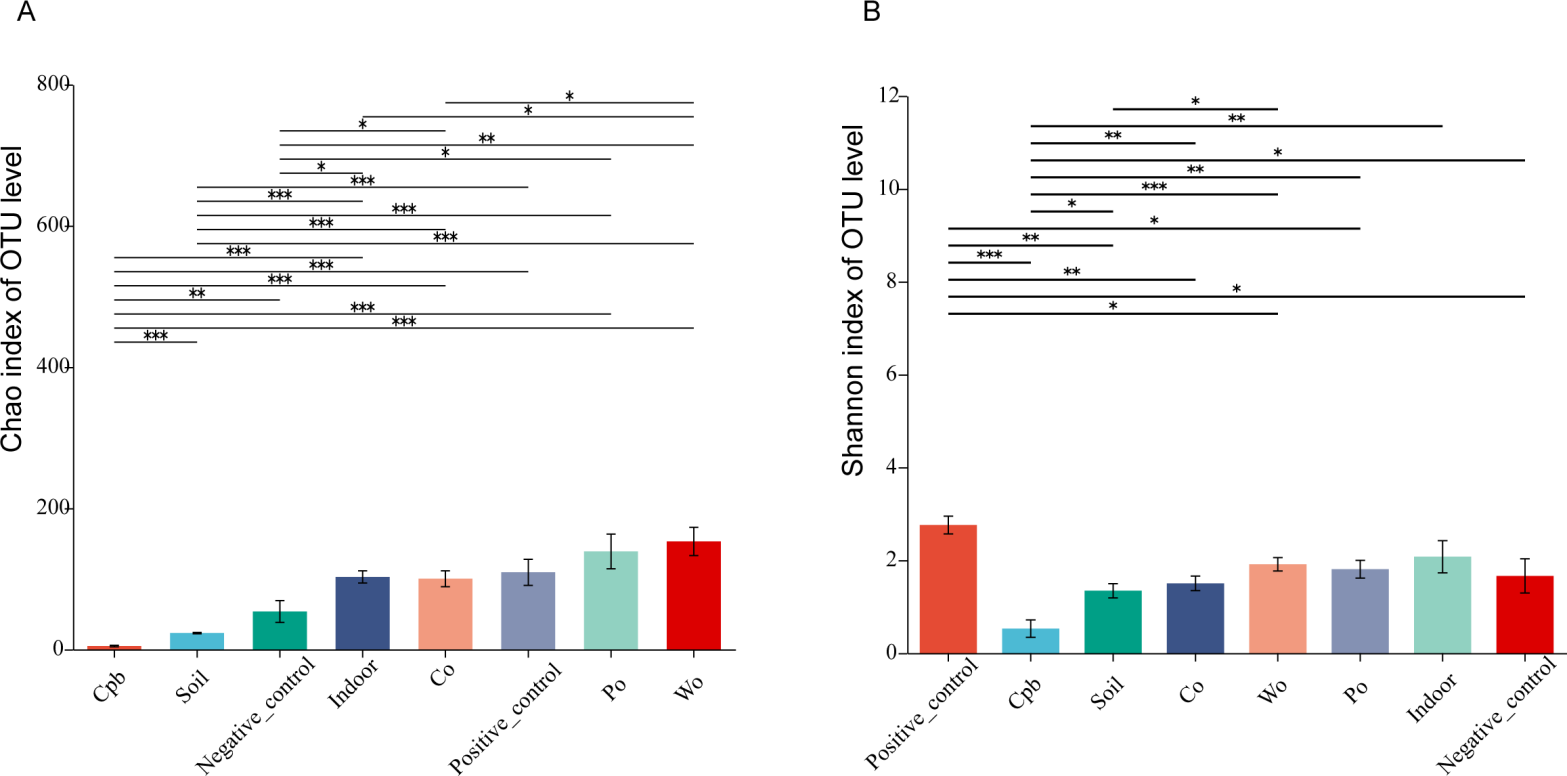
(**A**) Chao index and (**B**) Shannon index diagram of each group. “Cpb,” “soil,” “indoor,” “co,” “po,” “wo” represented the treatment group that exposed semen to the closed plastic bags, soil, indoor environment, the carriers of different materials (cotton, polyester, and wool fabric), respectively. (0.01< *P* ≤ 0.05 is marked as *, 0.001<*P* ≤ 0.01 is marked as **, *P* ≤ 0.001 is marked as ***)

According to the Chao index of each group, the index values of the closed plastic bag group, soil group, and negative control group were relatively low and were significantly different from the Chao indices of the other groups (*P* < 0.05) (Fig. 2A). According to the Shannon index, the index value of the positive control group was the highest, which was significantly different from that of the other groups (except for the indoor group) (*P* < 0.05), and the index value of the CPB group was the lowest (*P* < 0.05) (Fig. 2B). Similarly, the Shannon indices of the indoor exposure group, cotton group, polyester group, and wood fabric group were also similar (Table S2). That is to say, through Chao index, Shannon index, and their differences among groups, it can be revealed that the community richness and diversity of CPB group are the lowest, followed by the soil group. The highest diversity of the community was in the positive control group. There were no significant differences in community diversity among the indoor exposure group, cotton group, polyester group, and wool fabrics group.

### Compositional analysis of semen samples and significant difference analysis

**Fig 3:**
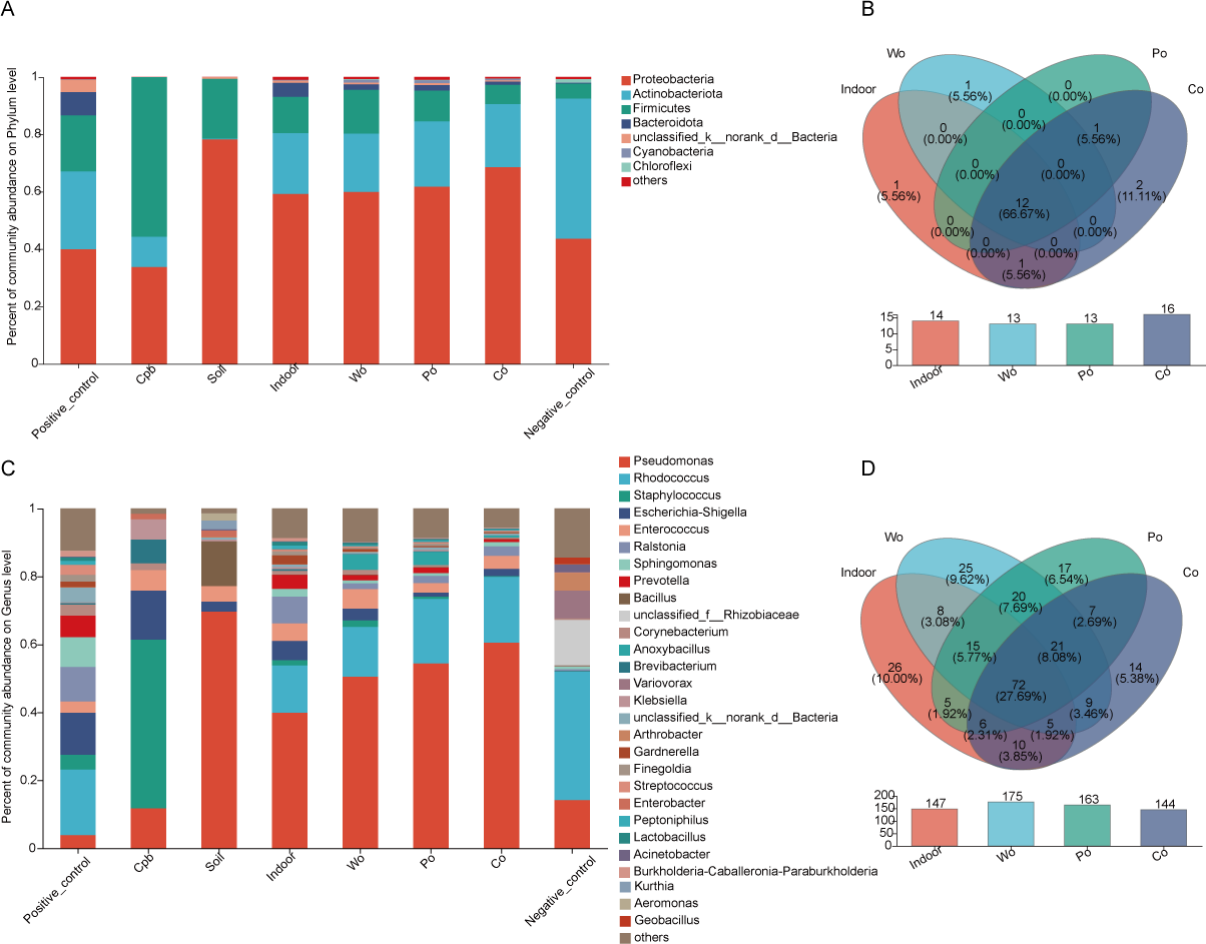
The mean relative abundances and Venn diagrams of bacterial phyla (**A, B**) and genera (**C, D**) represented in the V3-V4 16S rDNA amplicons obtained for samples from each groups.

The community barplot analysis at the phylum level showed that the microorganisms were mainly Proteobacteria, Actinobacteriota, and Firmicutes (Fig. S2A). Except for those of the CPB group and soil group, the microbial community compositions at the phylum level before and after semen exposure were similar, especially for those of the indoor group, cotton group, polyester group, and wood fabrics group (Fig. 3A). The Venn diagram analysis at the phylum level shows that there revealed 12 (66.67%) common phyla in the indoor group, cotton group, polyester group, and wood fabrics group (Fig. 3B).

The community barplot analysis at the genus level showed that the main microbial genera were *Pseudomonas*, *Rhodococcus*, *Staphylococcus*, *Shigella*, and *Enterococcus* (Fig. S2B). At the genus level, the relative abundance of *Pseudomonas* in the positive control group was low, but after the semen samples were exposed, the abundance of *Pseudomonas* increased significantly except in the CPB group, especially in the soil group. After the semen samples were exposed, the abundance of *Rhodococcus* changed significantly in CPB group and soil group, and its relative abundance decreased sharply to almost non-existence. In addition, the relative abundances of *Ralstonia*, *Sphingomonas,* and *Prevotella* decreased after exposure, especially in the CPB group and soil group. Compared with the positive control group, the relative abundance of *Staphylococcus* in the CPB group increased significantly to 49.61%, and the relative abundance of *Bacillus* in the soil group increased significantly to 13.14% (Fig. 3C). The Venn diagram analysis at the genus level shows that there revealed 72 (27.69%) common genera in the indoor group, cotton group, polyester group, and wood fabrics group (Fig. 3D).

**Fig 4:**
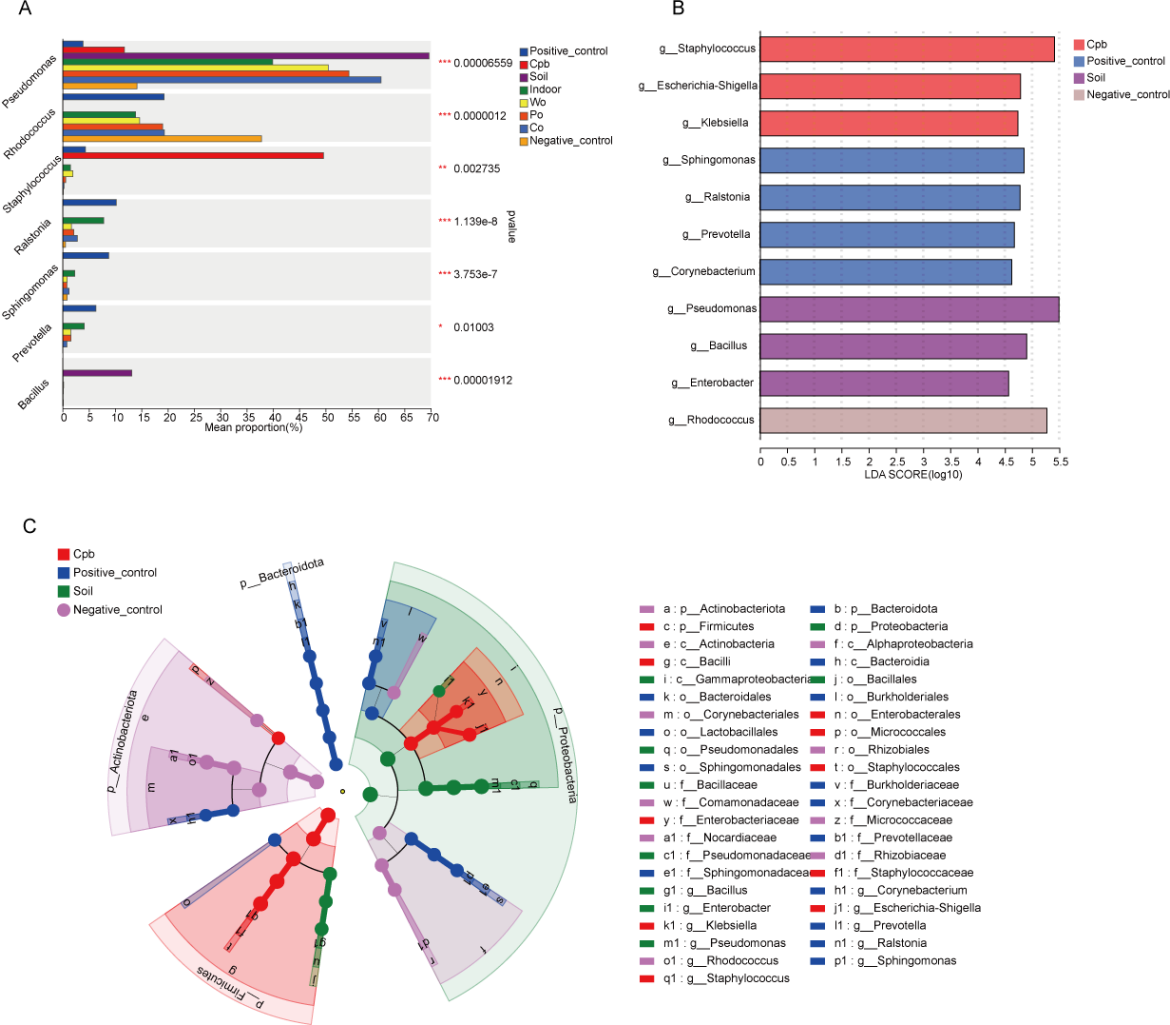
(**A**) The barplot displayed the differences in relative abundance of the same species among different groups. (**B, C**) The developmental tree diagram showed the differential microorganisms obtained at different levels of species hierarchy among different groups.

Based on the community abundance data in the sample, the Kruskal-Wallis *H* test was used to detect the species with different abundances in different microbial communities, carry out hypothesis tests, and evaluate the significance of the observed differences. The results of the Kruskal-Wallis *H* test barplot showed that the relative abundances of *Pseudomonas*, *Rhodococus*, *Staphylococus*, *Ralstonia*, *Sphingomonas*, *Prevotella,* and *Bacillus* had significant differences among the different groups (Fig. 4A).

Using the Lefse method, the microbial species with significant differences in the groups were screened. When the LDA threshold was set to 4, the indoor exposure group and the groups exposed to the three different carriers did not show any significantly enriched species. The species significantly enriched in the positive control group were *Ralstonia*, *Sphingomonas*, and *Prevotella; Rhodococcus* was significantly enriched in the negative control group; *Staphylococcus* was significantly enriched in the CPB group; and *Pseudomonas* and *Bacillus* were significantly enriched in the soil exposure group (Fig. 4B and C).

### Beta diversity analysis

Beta diversity analysis was used to perform comparative analysis among groups to explore the similarity or difference of community composition among samples in different groups.

**Fig 5:**
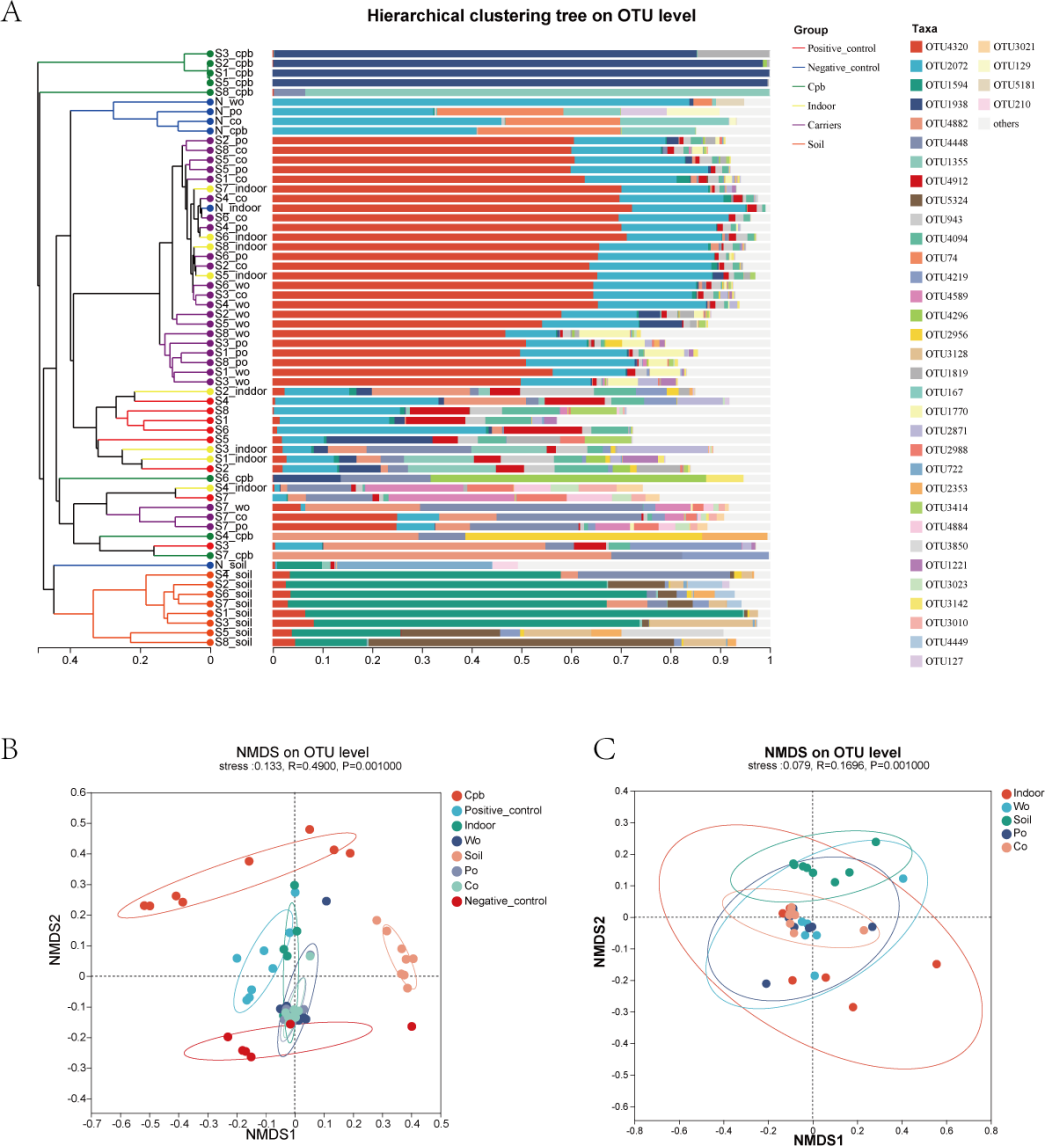
(**A**) Hierarchical clustering of the distance matrix can reveal the distance between sample branches, and samples can be divided into cohesive groups based on different distance thresholds. (**B, C**) The non-metric multidimensional scaling (NMDS) diagram of samples in the different exposure groups. Points of different colors represent samples of different groups, and the closer the two sample points are, the more similar the species composition of the two samples is.

Based on the Bray-Curtis distance algorithm, cluster analysis was carried out for all the samples. According to the results of the sample hierarchical clustering tree at the OTU level, it could be seen that the samples in the soil group could be clustered, some samples of the CPB group and the positive control group were clustered separately, and samples exposed to three different materials and some samples of the indoor group were clustered (Fig. 5A).

In non-metric multidimensional scaling (NMDS) analysis, points with different colors or shapes represent samples in different groups. The closer the two sample points are, the more similar the species composition of the two samples is. According to the results of NMDS at the OTU level, the samples of different groups could be roughly distinguished, but the separation of each group was not pronounced (Fig. 5B). Particularly, the semen samples subjected to exposure from three different materials exhibited substantial overlap and proved challenging to differentiate.(Fig. 5C).

### Differences among the microbial communities of the body fluids

**Fig 6:**
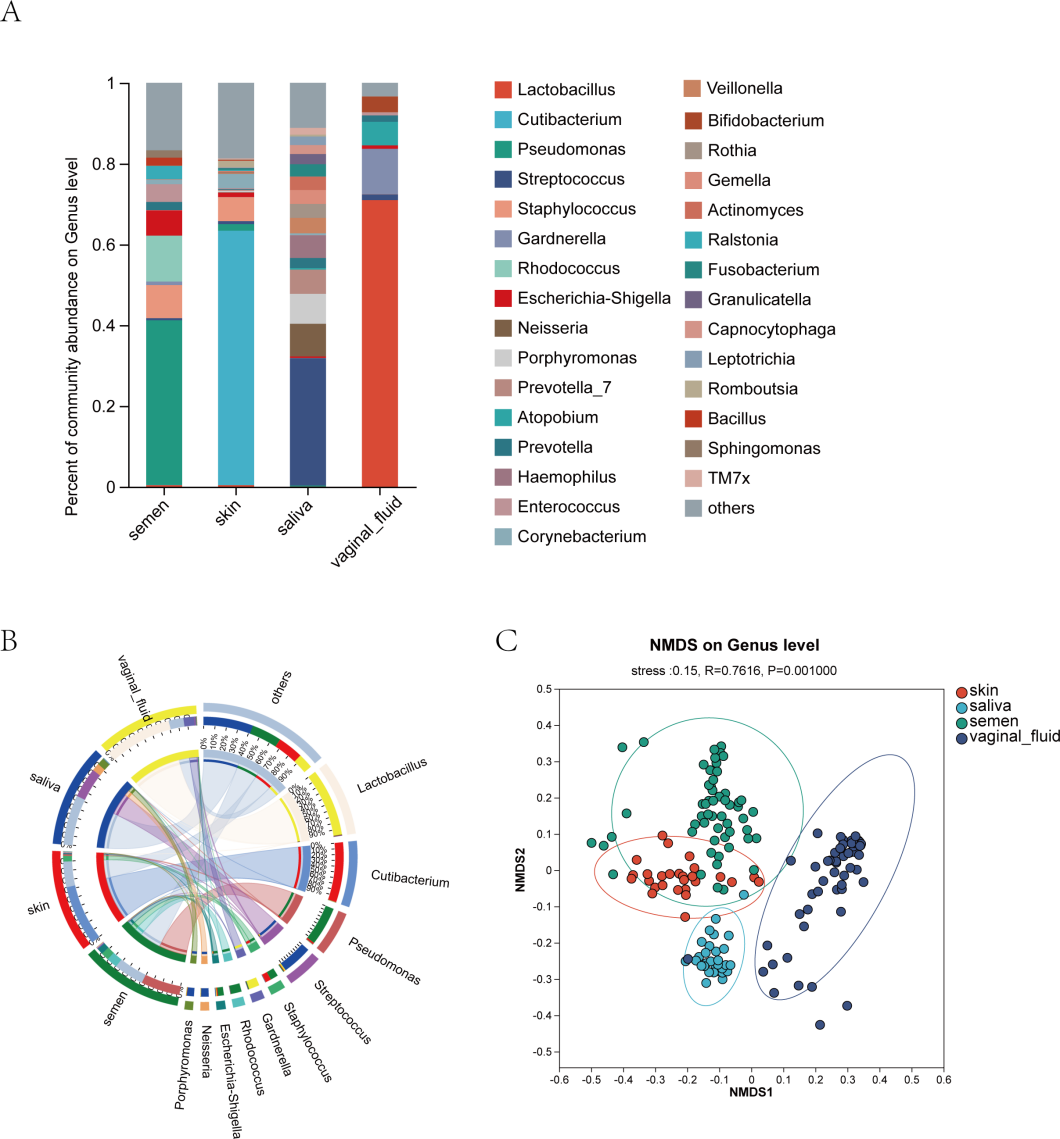
(**A**) Bar graph of microbial community composition of semen, skin, saliva, and vaginal fluid. (**B**) The Circos graph illustrated the proportional distribution of dominant genera within each group. (**C**) The non-metric multidimensional scaling diagram of samples in the different body fluids.

Comparing the microbiota of semen samples (both before and after exposure) with that of skin, saliva, and vaginal fluids from our previously published articles (11, 12), we found distinct variations in the microbial compositions of each bodily fluid (Fig. 6A). Specifically, the predominant bacterial genera were identified as *Pseudomonas* in semen, *Cutibacterium* in skin, *Streptococcus* in saliva, and *Lactobacillus* in vaginal fluids (Fig. 6B). The samples within each of these body fluids clustered cohesively, and there was evident discrimination between the different groups (Fig. 6C).

### Using random forest model to identify the body fluids

**Fig 7:**
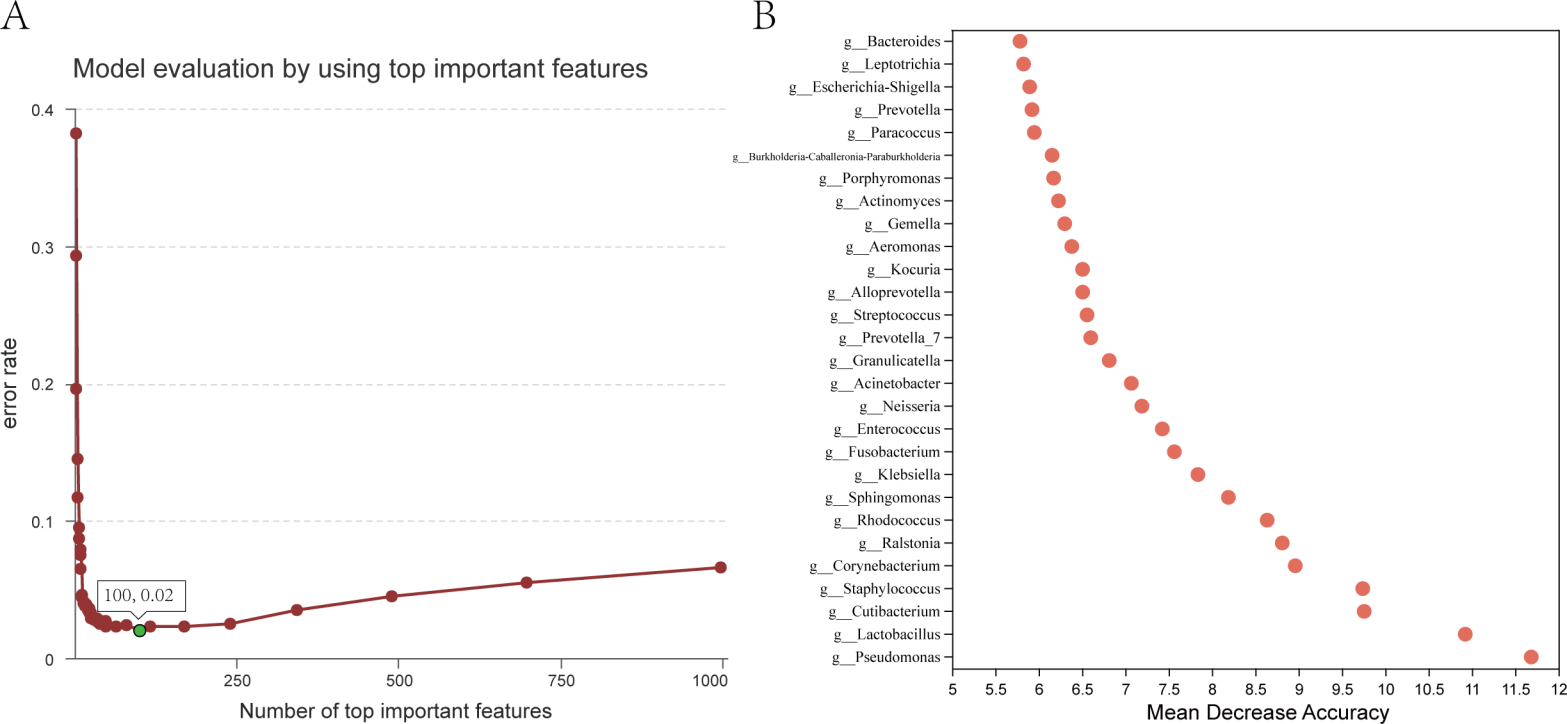
(**A**) Model evaluation by using top important features. The X-axis represents the number of top important features, and the Y-axis represents the average prediction error rate using 10-fold cross-validation. (**B**) Bubble plot of the mean decrease accuracy values of the top 30 important features.

Common samples in sexual assault cases include semen, skin and saliva, and vaginal secretions. Therefore, 27 skin samples, 30 saliva samples, and 66 vaginal fluid samples were selected for analysis together with 56 semen samples in this study. According to the results of 10-fold cross-validation, when the top 100 important features were selected at least, the prediction error rate of this model was the lowest, which was 0.02 (Fig. 7A). The prominent features that significantly contributed to the model’s performance included genera such as *Pseudomonas*, *Lactobacillus*, *Cutibacterium*, *Corynebacterium*, *Staphylococcus*, among others (Fig. 7B). The random forest model selected the top 100 important features and obtained the distribution probability value of the validation sample. All 53 samples in the validation set could be correctly classified, and the correctness rate reached 100% (Table 1).

**TABLE 1:** The prediction results of the random forest classification model on 53 test samples, including the predicted grouping and the distribution probability of samples^*a*^

Sample name	Real grouping	Predict grouping	Saliva probability	Semen probability	Skin probability	Vaginal fluid probability
S6B	Saliva	Saliva	**0.954**	0.012	0.026	0.008
S7B	Saliva	Saliva	**0.904**	0.018	0.032	0.046
S8B	Saliva	Saliva	**0.9**	0.026	0.036	0.038
S4B	Saliva	Saliva	**0.846**	0.022	0.07	0.062
S4_B	Saliva	Saliva	**0.844**	0.024	0.076	0.056
S9B	Saliva	Saliva	**0.826**	0.022	0.092	0.06
S3_B	Saliva	Saliva	**0.772**	0.088	0.066	0.074
S5B	Saliva	Saliva	**0.724**	0.036	0.09	0.15
S3B	Saliva	Saliva	**0.578**	0.064	0.192	0.166
S2	Semen	Semen	0	**0.944**	0.04	0.016
S1_co	Semen	Semen	0.002	**0.94**	0.042	0.016
S1_po	Semen	Semen	0.002	**0.896**	0.03	0.072
S6_po	Semen	Semen	0	**0.884**	0.064	0.052
S4_po	Semen	Semen	0.008	**0.874**	0.05	0.068
S6_outdoor	Semen	Semen	0.002	**0.872**	0.05	0.076
S7_outdoor	Semen	Semen	0.006	**0.846**	0.068	0.08
S2_co	Semen	Semen	0.006	**0.838**	0.06	0.096
S4	Semen	Semen	0.004	**0.838**	0.072	0.086
S8_outdoor	Semen	Semen	0.004	**0.836**	0.066	0.094
S5_outdoor	Semen	Semen	0	**0.802**	0.09	0.108
S8_soil	Semen	Semen	0.002	**0.794**	0.04	0.164
S6_soil	Semen	Semen	0.006	**0.786**	0.034	0.174
S7	Semen	Semen	0.036	**0.786**	0.078	0.1
S4_soil	Semen	Semen	0.004	**0.702**	0.026	0.268
S5_indoor	Semen	Semen	0	**0.622**	0.04	0.338
S1	Semen	Semen	0.08	**0.548**	0.336	0.036
S7A	Skin	Skin	0.058	0.112	**0.806**	0.024
S6A	Skin	Skin	0.032	0.104	**0.8**	0.064
S4A	Skin	Skin	0.046	0.118	**0.798**	0.038
S8A	Skin	Skin	0.06	0.124	**0.658**	0.158
S5A	Skin	Skin	0.14	0.168	**0.618**	0.074
S3_A	Skin	Skin	0.008	0.404	**0.462**	0.126
S4_A	Skin	Skin	0.038	0.314	**0.442**	0.206
S3A	Skin	Skin	0.004	0.294	**0.394**	0.308
Y11_gd	Vaginal fluid	Vaginal fluid	0	0	0	**1**
Y11_hn	Vaginal fluid	Vaginal fluid	0	0	0	**1**
Y12_hn	Vaginal fluid	Vaginal fluid	0	0	0	**1**
Y15_gd	Vaginal fluid	Vaginal fluid	0	0	0	**1**
Y22_gd	Vaginal fluid	Vaginal fluid	0	0	0	**1**
y14_xj	Vaginal fluid	Vaginal fluid	0	0	0	**1**
Y7_gd	Vaginal fluid	Vaginal fluid	0	0	0.004	**0.996**
Y7_hn	Vaginal fluid	Vaginal fluid	0.002	0.006	0.01	**0.982**
Y5_gd	Vaginal fluid	Vaginal fluid	0	0.038	0	**0.962**
Y18_gd	Vaginal fluid	Vaginal fluid	0	0.09	0.024	**0.886**
y19_xj	Vaginal fluid	Vaginal fluid	0	0.082	0.056	**0.862**
y20_xj	Vaginal fluid	Vaginal fluid	0.016	0.13	0.002	**0.852**
y6_xj	Vaginal fluid	Vaginal fluid	0.024	0.096	0.032	**0.848**
Y17_gd	Vaginal fluid	Vaginal fluid	0	0.158	0.006	**0.836**
y12_xj	Vaginal fluid	Vaginal fluid	0	0.138	0.03	**0.832**
Y23_gd	Vaginal fluid	Vaginal fluid	0	0.166	0.012	**0.822**
y10_xj	Vaginal fluid	Vaginal fluid	0.132	0.042	0.032	**0.794**
Y20_gd	Vaginal fluid	Vaginal fluid	0	0.288	0.062	**0.65**
y22_xj	Vaginal fluid	Vaginal fluid	0.016	0.338	0.036	**0.61**

^*a*^ The bold part indicated the highest distribution probability.

## DISCUSSION

Several studies have shown that semen microbes can be used to identify body fluids but few studies on semen exposure. Moreover, the existing semen exposure research is relatively simple, which is not enough to meet the needs of forensic practice. This study was designed to simulate different situations of semen exposure in sexual crime cases for further research and revealed the changes of semen microbiota under different environmental exposure through 16S rRNA gene sequencing.

The dominant phyla in the positive control group were Proteobacteria (40%), Actinobacteriota (27%), Firmicutes (20%), and Bacteroidetes (8%), which were similar to the findings of the semen samples of healthy men in the studies of Chen et al. (10) and Yang et al. (13). In the positive control group, the relative abundances of *Rhodococcus*, *Escherichia-Shigella*, *Ralstonia*, *Sphingomonas,* and *Prevotella* at the genus level were high, which is slightly different from the findings of other studies (10, 14, 15). However, Hou et al. observed that the species composition of semen communities varied widely among men, suggesting that each individual had unique and perhaps personalized bacterial communities in their semen (14). As observed in the research results, seminal microbiota communities were significantly more diverse than vaginal microbial communities were, and there were no predominant microorganisms in most semen samples (16). Studies have shown that there is *Lactobacillus* in semen, the most abundant genera of bacteria are *Lactobacillus* (19.9%) (15), and the OTU number of *Lactobacillus* was 6.79% (10) in the healthy male group, which is different from our results (1%). *Lactobacillus* also did not make up a high relative abundance of the semen microbiome in Dobay’s study (8).

In addition, a large number of lactobacilli have been detected in female vaginal secretions (17), and sexual intercourse between men and women can cause changes in the semen microbiome (14). Mändar et al. found that the composition of semen microorganisms was differed among men with asexual experience (18). It is possible that most of the volunteers in this study had no sexual experience, so there was no transfer between microorganisms. Therefore, only a small amount of *Lactobacillus* was present in some semen samples of the positive control group.

In real crime scenes, semen samples are always exposed to microbe-laden environments, and it is critical to consider the relationship between semen microbes and environmental microbes. Semen samples collected at crime scenes may have been left in a variety of settings for a period before being discovered, such as on fabrics like clothing and bed sheets, within sealed garbage bags, or even in the soil. In view of this, this study simulated the possible exposure scenarios of samples encountered at crime scenes and explored the changing characteristics of semen microbial community under different fabric materials, closed packaging, and multi-microbial background, aiming to provide a more scientific and rigorous analysis basis for forensic investigations.

The results showed that the community diversity of each exposure group decreased significantly compared with that of the positive control group, which is similar to the findings of previous studies (9). Special attention should be given to the closed plastic bags and soil environments. The community richness and diversity of the CPB group were the lowest, followed by the soil group. These two special environmental factors may lead to changes in semen microorganisms, which are not conducive to the growth of microbial species and quantity. The LEfSe results showed that *Staphylococcus* in the CPB group was significantly enriched, while *Pseudomonas* and *Bacillus* were significantly enriched in the soil exposure group. The CPB group and soil group showed a trend from high diversity to dominated by several dominant bacteria. This suggests that in actual cases, when semen is put in a closed plastic bag by a suspect or exposed to the soil environment, the microbial community will change significantly and will be dominated by the microorganisms that adapt to the environment, and these microorganisms will become the dominant bacteria. This approach may have a suggestive effect on the prediction of the environment of the crime scene where the semen samples come from.

In addition, several bacterial genera with obvious changes deserve attention. After exposure of semen samples, the abundance of *Pseudomonas* increased significantly except for CPB group, especially in the soil group. *Rhodococcus* had obvious changes in the CPB group and soil group, and its abundance decreased sharply. In addition, the abundances of *Ralstonia*, *Sphingomonas,* and *Prevotella* decreased after exposure, especially in the CPB group and soil group. As a highly adaptable symbiotic organism, the ability of *Staphylococcus* to thrive under anaerobic conditions is particularly significant (19). After semen exposure to the CPB group, the relative abundance of *Staphylococcus* increased significantly to 49.61%, and after exposure to the soil group, the relative abundance of *Bacillus* increased significantly to 13.14%. *Bacillus* is a kind of aerobic or facultative anaerobic bacteria, and most of which are saprophytic bacteria (20). *Bacillus* species are ubiquitous in nature, mainly distributed in soil, plant surface, and water, with highly resistant spores, so they can tolerate a variety of adverse environments (21). The relative abundance of the above bacteria was significantly different among the different groups.

There was no significant difference in community diversity among the indoor exposure group, cotton group, polyester group, and wool fabrics group. The Venn diagram analysis at the phylum level showed that the common phyla in the indoor group, cotton group, polyester group, and wool fabrics group are up to 66.67%. According to the results of the hierarchical clustering tree at the OTU level, the samples exposed to three different materials and some samples from the indoor group were clustered. The NMDS analysis diagram also showed that the sample distance between each group was too short to distinguish between each group of samples. The above results showed that whether the sample is exposed to the carrier and what kind of carrier it is exposed to had no obvious impact on the semen microorganism.

While the NMDS analysis at the OTU level permitted a general discrimination among samples from differing exposure groups, the distinction between each group was not obvious. Furthermore, every body fluid possesses a distinctive microbial community structure, with dominant bacterial genera playing a pivotal role in distinguishing among various bodily fluids. Notably, genera such as *Pseudomonas*, *Lactobacillus*, *Cutibacterium*, *Corynebacterium*, and *Staphylococcus* contributed significantly to this differentiation. Based on the above analysis, a random forest model was devised to differentiate between semen exposed to environmental conditions and three normal tissues and body fluids. The model achieved a 100% accuracy rate in recognizing semen. It indicated that when semen was exposed to a specific environment, its microbiota could still maintain a certain stability, and semen could still be identified by a random forest model. Overall, RF model based on 16S rRNA gene sequencing had good application potential for body fluid identification.

At present, the results are very exciting, but there are several limitations that need to be acknowledged. The sample has only undergone exposure for a period of 15 days, and extending the duration of exposure is an area that merits further investigation. The sample was only exposed for 15 days. In addition, more exposure situations are worth simulating for further research to be closer to the actual case.

### Conclusion

In this study, high-throughput sequencing was used to observe the changes in the microbial community after 15 days of semen exposure under different forensic simulation conditions. The results showed that the microorganism in the closed plastic bag and soil exposure changed significantly, and the microbial community was dominated by the bacteria adapted to the environment, while the other exposure groups did not change significantly. In addition, compared with saliva, skin, and vaginal fluid reported previously, the exposed semen was still recognized correctly as semen. In conclusion, the semen microbiome seems promising for fluid recognition, even after semen exposure.

## Data Availability

The raw data were uploaded to the NCBI SRA database under accession number: PRJNA1102678.

## SUPPLEMENTAL MATERIAL

Fig. S1 spectrum.00125-24-s0001.tif Shannon curves.

Fig. S2 spectrum.00125-24-s0002.tif Relative abundances of bacterial phyla (A) and genera (B) for each sample.

Supplemental material spectrum.00125-24-s0003.docx Tables S1 and S2.

ASM does not own the copyrights to Supplemental Material that may be linked to, or accessed through, an article. The authors have granted ASM a non-exclusive, world-wide license to publish the Supplemental Material files. Please contact the corresponding author directly for reuse.

## References

[1] Soares-Vieira JA, Billerbeck AEC, Iwamura ESM, Zampieri RA, Gattás GJF, Munoz DR, Hallak J, Mendonca BB, Lucon AM. 2007. Y-STRs in forensic medicine: DNA analysis in semen samples of azoospermic individuals. J Forensic Sci 52:664–670. doi:10.1111/j.1556-4029.2007.00433.x 17456093

[2] Martínez P, Santiago B, Alcalá B, Atienza I. 2015. Semen searching when sperm is absent. Sci Justice 55:118–123. doi:10.1016/j.scijus.2015.01.008 25753997

[3] Forat S, Huettel B, Reinhardt R, Fimmers R, Haidl G, Denschlag D, Olek K. 2016. Methylation markers for the identification of body fluids and tissues from forensic trace evidence. PLoS One 11:e0147973. doi:10.1371/journal.pone.0147973 26829227 PMC4734623

[4] Sakurada K, Watanabe K, Akutsu T. 2020. Current methods for body fluid identification related to sexual crime focusing on saliva, semen, and vaginal fluid. Diagnostics (Basel) 10:693. doi:10.3390/diagnostics10090693 32937964 PMC7555023

[5] Christensen BC, Houseman EA, Marsit CJ, Zheng S, Wrensch MR, Wiemels JL, Nelson HH, Karagas MR, Padbury JF, Bueno R, Sugarbaker DJ, Yeh RF, Wiencke JK, Kelsey KT. 2009. Aging and environmental exposures alter tissue-specific DNA methylation dependent upon CpG island context. PLoS Genet 5:e1000602. doi:10.1371/journal.pgen.1000602 19680444 PMC2718614

[6] Sijen T. 2015. Molecular approaches for forensic cell type identification: on mRNA, miRNA, DNA methylation and microbial markers. Forensic Sci Int Genet 18:21–32. doi:10.1016/j.fsigen.2014.11.015 25488609

[7] Tackmann J, Arora N, Schmidt TSB, Rodrigues JFM, von Mering C. 2018. Ecologically informed microbial biomarkers and accurate classification of mixed and unmixed samples in an extensive cross-study of human body sites. Microbiome 6:192. doi:10.1186/s40168-018-0565-6 30355348 PMC6201589

[8] Dobay A, Haas C, Fucile G, Downey N, Morrison HG, Kratzer A, Arora N. 2019. Microbiome-based body fluid identification of samples exposed to indoor conditions. Forensic Sci Int Genet 40:105–113. doi:10.1016/j.fsigen.2019.02.010 30785061

[9] Yao T, Han X, Guan T, Wang Z, Zhang S, Liu C, Liu C, Chen L. 2020. Effect of indoor environmental exposure on seminal microbiota and its application in body fluid identification. Forensic Sci Int 314:110417. doi:10.1016/j.forsciint.2020.110417 32702532

[10] Chen H, Luo T, Chen T, Wang G. 2018. Seminal bacterial composition in patients with obstructive and non-obstructive azoospermia. Exp Ther Med 15:2884–2890. doi:10.3892/etm.2018.5778 29456693 PMC5795641

[11] Yao T, Han X, Guan T, Zhai C, Liu C, Liu C, Zhu B, Chen L. 2021. Exploration of the microbiome community for saliva, skin, and a mixture of both from a population living in Guangdong. Int J Legal Med 135:53–62. doi:10.1007/s00414-020-02329-6 32583081

[12] Yao T, Wang Z, Liang X, Liu C, Yu Z, Han X, Liu R, Liu Y, Liu C, Chen L. 2021. Signatures of vaginal microbiota by 16S rRNA gene: potential bio-geographical application in Chinese Han from three regions of China. Int J Legal Med 135:1213–1224. doi:10.1007/s00414-021-02525-y 33594458

[13] Yang H, Zhang J, Xue Z, Zhao C, Lei L, Wen Y, Dong Y, Yang J, Zhang L. 2020. Potential pathogenic bacteria in seminal microbiota of patients with different types of dysspermatism. Sci Rep 10:6876. doi:10.1038/s41598-020-63787-x 32327694 PMC7181748

[14] Hou D, Zhou X, Zhong X, Settles ML, Herring J, Wang L, Abdo Z, Forney LJ, Xu C. 2013. Microbiota of the seminal fluid from healthy and infertile men. Fertil Steril 100:1261–1269. doi:10.1016/j.fertnstert.2013.07.1991 23993888 PMC3888793

[15] Weng SL, Chiu CM, Lin FM, Huang WC, Liang C, Yang T, Yang TL, Liu CY, Wu WY, Chang YA, Chang TH, Huang HD. 2014. Bacterial communities in semen from men of infertile couples: metagenomic sequencing reveals relationships of seminal microbiota to semen quality. PLoS One 9:e110152. doi:10.1371/journal.pone.0110152 25340531 PMC4207690

[16] Mändar Reet, Punab M, Borovkova N, Lapp E, Kiiker R, Korrovits P, Metspalu A, Krjutškov K, Nõlvak H, Preem J-K, Oopkaup K, Salumets A, Truu J. 2015. Complementary seminovaginal microbiome in couples. Res Microbiol 166:440–447. doi:10.1016/j.resmic.2015.03.009 25869222

[17] Koedooder R, Mackens S, Budding A, Fares D, Blockeel C, Laven J, Schoenmakers S. 2019. Identification and evaluation of the microbiome in the female and male reproductive tracts. Hum Reprod Update 25:298–325. doi:10.1093/humupd/dmy048 30938752

[18] Mändar R, Türk S, Korrovits P, Ausmees K, Punab M. 2018. Impact of sexual debut on culturable human seminal microbiota. Andrology 6:510–512. doi:10.1111/andr.12482 29512338

[19] Hall JW, Ji Y. 2013. Sensing and adapting to anaerobic conditions by Staphylococcus aureus. Adv Appl Microbiol 84:1–25. doi:10.1016/B978-0-12-407673-0.00001-1 23763757

[20] Patiño-Navarrete R, Sanchis V. 2017. Evolutionary processes and environmental factors underlying the genetic diversity and lifestyles of Bacillus cereus group bacteria. Res Microbiol 168:309–318. doi:10.1016/j.resmic.2016.07.002 27424810

[21] Maughan H, Van der Auwera G. 2011. Bacillus taxonomy in the genomic era finds phenotypes to be essential though often misleading. Infect Genet Evol 11:789–797. doi:10.1016/j.meegid.2011.02.001 21334463

