# Supplementary figures and images for: Changes in the microbial community of semen exposed to different simulated forensic situations
Source: Microbiol Spectr. 2024 Jul 9;12(8):e00125-24. doi: 10.1128/spectrum.00125-24 (PMC11302308; doi:10.1128/spectrum.00125-24)

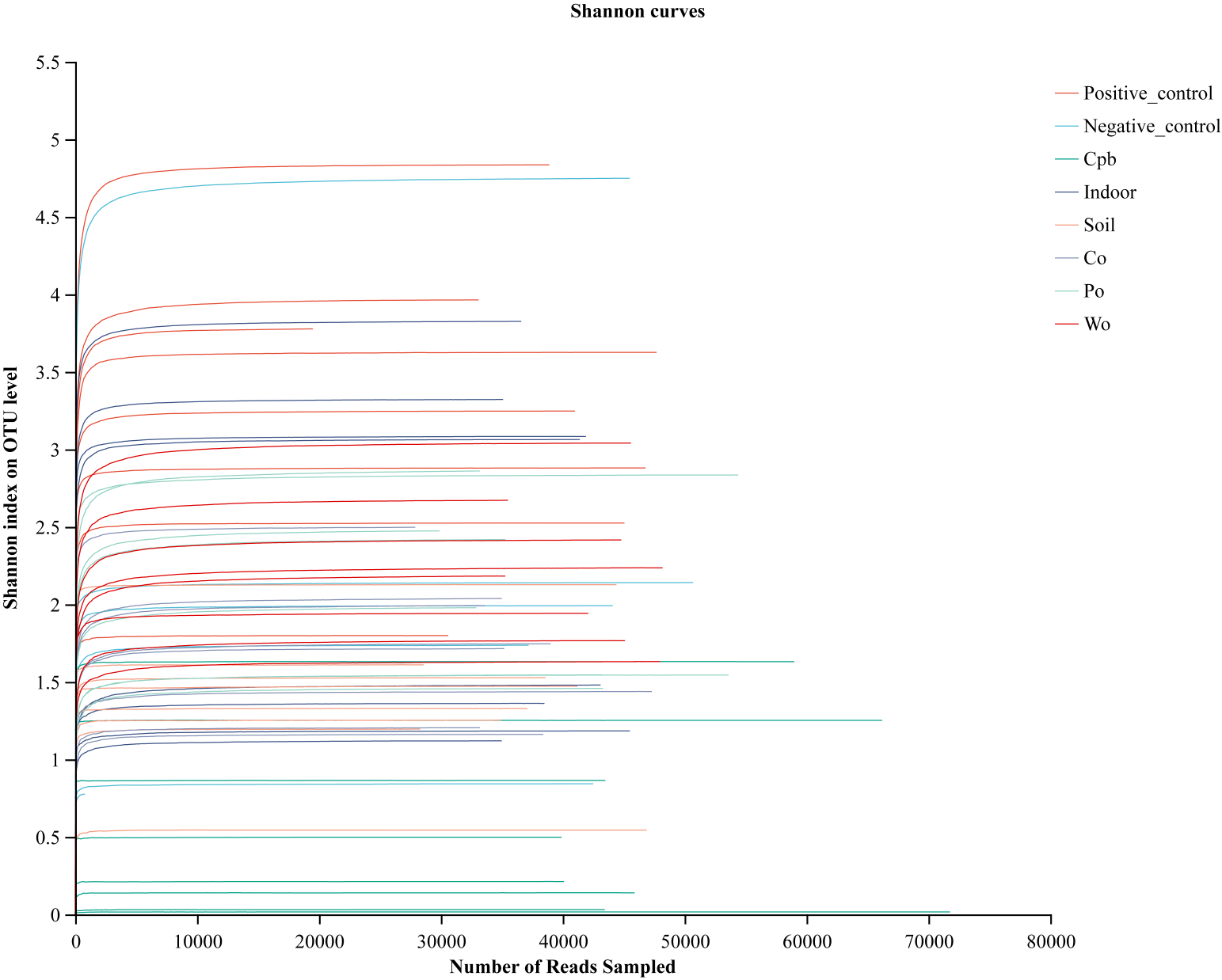

Supplement: Fig. S1 — Shannon curves. [file spectrum.00125-24-s0001.tif]

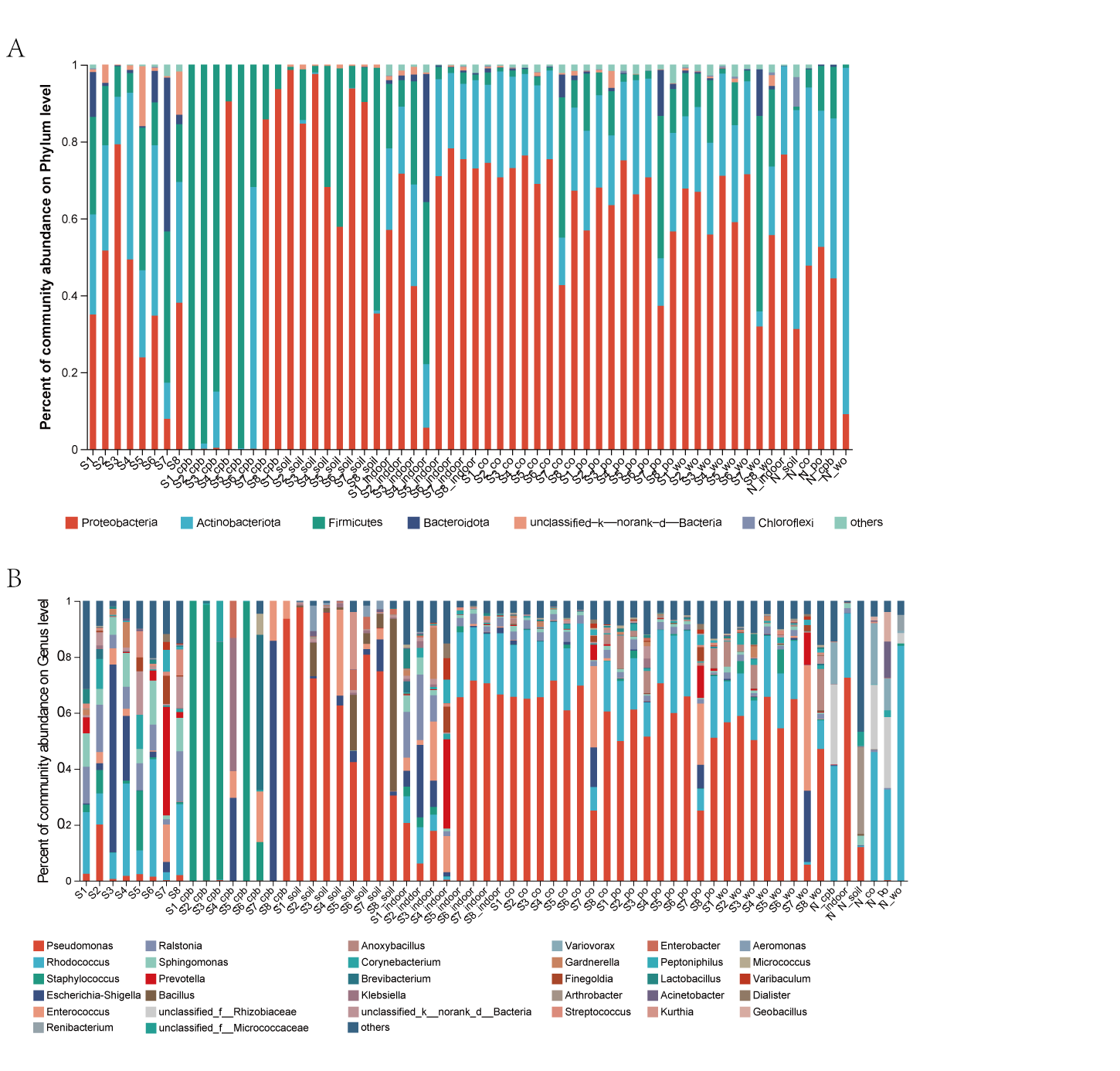

Supplement: Fig. S2 — Relative abundances of bacterial phyla (A) and genera (B) for each sample. [file spectrum.00125-24-s0002.tif]
