# Supplementary material for: Changes in the microbial community of semen exposed to different simulated forensic situations
Source: Microbiol Spectr. 2024 Jul 9;12(8):e00125-24. doi: 10.1128/spectrum.00125-24 (PMC11302308; doi:10.1128/spectrum.00125-24)
Supplement: Supplemental material — Tables S1 and S2. [file spectrum.00125-24-s0003.docx]

Figure S1 The end of the Shannon curves tends to be flat, indicating a reasonable amount of sequencing data.

Figure S2 The relative abundances of bacterial phyla (A) and genera (B) for each sample.

Table S1 Sample sequencing details for this study.

| Sample\Info | Sequence number | Base number | Mean length | Min_length | Max_length |
| --- | --- | --- | --- | --- | --- |
| N_co | 38116 | 15749095 | 413.188556 | 399 | 432 |
| N_cpb | 50136 | 20581833 | 410.520045 | 345 | 479 |
| N_indoor | 42767 | 18113719 | 423.544298 | 332 | 491 |
| N_po | 53523 | 22238090 | 415.486613 | 262 | 498 |
| N_soil | 70328 | 29128596 | 414.182061 | 210 | 458 |
| N_wo | 772 | 316390 | 409.831606 | 399 | 429 |
| S1 | 45196 | 18823384 | 416.483406 | 200 | 513 |
| S1_co | 35931 | 15177023 | 422.39356 | 200 | 534 |
| S1_cpb | 72476 | 31091134 | 428.985236 | 404 | 430 |
| S1_indoor | 38208 | 15990730 | 418.51785 | 200 | 536 |
| S1_po | 30872 | 13027498 | 421.984258 | 200 | 534 |
| S1_soil | 49192 | 21102092 | 428.974061 | 380 | 432 |
| S1_wo | 49773 | 21055035 | 423.021216 | 200 | 526 |
| S2 | 41734 | 17198862 | 412.106724 | 200 | 530 |
| S2_co | 48001 | 20259024 | 422.054207 | 200 | 534 |
| S2_cpb | 46926 | 20107679 | 428.497613 | 283 | 431 |
| S2_inddor | 36917 | 15489277 | 419.570306 | 200 | 516 |
| S2_po | 33537 | 14135402 | 421.486776 | 200 | 524 |
| S2_soil | 49086 | 21054561 | 428.932099 | 250 | 431 |
| S2_wo | 36366 | 15345404 | 421.971182 | 201 | 517 |
| S3 | 33168 | 14099803 | 425.102599 | 200 | 531 |
| S3_co | 39887 | 16852849 | 422.514829 | 202 | 518 |
| S3_cpb | 40750 | 17350280 | 425.773742 | 222 | 431 |
| S3_indoor | 42193 | 17625433 | 417.733581 | 200 | 522 |
| S3_po | 34058 | 14151788 | 415.52023 | 200 | 526 |
| S3_soil | 29996 | 12860165 | 428.729331 | 207 | 504 |
| S3_wo | 36491 | 15386641 | 421.655778 | 207 | 532 |
| S4 | 46040 | 19048810 | 413.744787 | 201 | 537 |
| S4_co | 33784 | 14307706 | 423.505387 | 355 | 432 |
| S4_cpb | 70590 | 30282442 | 428.990537 | 304 | 431 |
| S4_indoor | 42731 | 17875311 | 418.321851 | 200 | 520 |
| S4_po | 44069 | 18649023 | 423.177812 | 217 | 449 |
| S4_soil | 43008 | 18450029 | 428.99063 | 404 | 518 |
| S4_wo | 48906 | 20645819 | 422.15309 | 206 | 534 |
| S5 | 33833 | 13117765 | 387.721012 | 200 | 540 |
| S5_co | 35937 | 15160169 | 421.85405 | 200 | 524 |
| S5_cpb | 43954 | 18852261 | 428.908882 | 404 | 431 |
| S5_indoor | 46032 | 19471264 | 422.994091 | 200 | 469 |
| S5_po | 54537 | 23010731 | 421.928801 | 249 | 487 |
| S5_soil | 51094 | 21923892 | 429.089365 | 365 | 540 |
| S5_wo | 46016 | 19319035 | 419.832993 | 200 | 539 |
| S6 | 49618 | 20488841 | 412.931618 | 202 | 517 |
| S6_co | 38944 | 16495485 | 423.569356 | 261 | 453 |
| S6_cpb | 69471 | 28902944 | 416.043299 | 382 | 507 |
| S6_indoor | 35442 | 15021291 | 423.827408 | 201 | 534 |
| S6_po | 44136 | 18647696 | 422.505347 | 208 | 494 |
| S6_soil | 41391 | 17754104 | 428.936339 | 374 | 430 |
| S6_wo | 46337 | 19595459 | 422.890109 | 214 | 516 |
| S7 | 47240 | 19761960 | 418.331075 | 200 | 529 |
| S7_co | 28584 | 12107452 | 423.574447 | 202 | 524 |
| S7_cpb | 55952 | 24002767 | 428.988544 | 403 | 432 |
| S7_indoor | 43689 | 18420932 | 421.637758 | 200 | 540 |
| S7_po | 55541 | 23453393 | 422.271709 | 200 | 539 |
| S7_soil | 37878 | 16251189 | 429.040314 | 403 | 431 |
| S7_wo | 44685 | 19055064 | 426.430883 | 200 | 493 |
| S8 | 19938 | 7880083 | 395.229361 | 200 | 531 |
| S8_co | 34309 | 14456442 | 421.360051 | 200 | 535 |
| S8_cpb | 40854 | 17525771 | 428.985436 | 229 | 477 |
| S8_indoor | 39096 | 16528054 | 422.755627 | 202 | 506 |
| S8_po | 36323 | 15315492 | 421.647221 | 200 | 508 |
| S8_soil | 45925 | 19719363 | 429.381884 | 324 | 478 |
| S8_wo | 47034 | 19749943 | 419.90779 | 200 | 531 |

Table S2 Chao index and Shannon index of each group.

| Estimators | Chao (mean ± sd) | Shannon (mean ± sd) |
| --- | --- | --- |
| Co | 100.17 ± 31.82 | 1.4994 ± 0.44 |
| Positive_control | 109.19 ± 52.12 | 2.7547 ± 0.54 |
| Negative_control | 53.691 ± 37.92 | 1.6589 ± 0.90 |
| Wo | 152.97 ± 56.57 | 1.9103 ± 0.41 |
| Soil | 23.177 ± 2.70 | 1.34 ± 0.43 |
| Cpb | 4.8125 ± 3.29 | 0.52447 ± 0.53 |
| Indoor | 102.66 ± 24.47 | 2.0731 ± 0.98 |
| Po | 138.77 ± 69.20 | 1.8044 ± 0.54 |
| P_value(Co-Positive_control) | 0.7132 | 0.002762 |
| P_adjust(Co-Positive_control) | 0.8333 | 0.008285 |
| P_value(Negative_control-Positive_control) | 0.06121 | 0.04539 |
| P_adjust(Negative_control-Positive_control) | 0.122 | 0.122 |
| P_value(Positive_control-Wo) | 0.2271 | 0.01008 |
| P_adjust(Positive_control-Wo) | 0.2726 | 0.03025 |
| P_value(Positive_control-Soil) | 0.0009069 | 0.001359 |
| P_adjust(Positive_control-Soil) | 0.001631 | 0.001631 |
| P_value(Cpb-Positive_control) | 0.0009229 | 0.0009391 |
| P_adjust(Cpb-Positive_control) | 0.0009391 | 0.0009391 |
| P_value(Indoor-Positive_control) | 0.7929 | 0.1563 |
| P_adjust(Indoor-Positive_control) | 0.9162 | 0.4688 |
| P_value(Po-Positive_control) | 0.4309 | 0.01008 |
| P_adjust(Po-Positive_control) | 0.5171 | 0.03025 |
| P_value(Co-Negative_control) | 0.03316 | 0.7469 |
| P_adjust(Co-Negative_control) | 0.07731 | 0.7469 |
| P_value(Co-Wo) | 0.03132 | 0.08312 |
| P_adjust(Co-Wo) | 0.09168 | 0.1247 |
| P_value(Co-Soil) | 0.0009069 | 0.6365 |
| P_adjust(Co-Soil) | 0.001409 | 0.7638 |
| P_value(Co-Cpb) | 0.0009229 | 0.005385 |
| P_adjust(Co-Cpb) | 0.001396 | 0.006462 |
| P_value(Co-Indoor) | 0.7929 | 0.4309 |
| P_adjust(Co-Indoor) | 0.9161 | 0.9161 |
| P_value(Co-Po) | 0.4309 | 0.2701 |
| P_adjust(Co-Po) | 0.5171 | 0.4394 |
| P_value(Negative_control-Wo) | 0.005509 | 0.4014 |
| P_adjust(Negative_control-Wo) | 0.01626 | 0.4817 |
| P_value(Negative_control-Soil) | 0.1733 | 0.4014 |
| P_adjust(Negative_control-Soil) | 0.3467 | 0.4014 |
| P_value(Cpb-Negative_control) | 0.002362 | 0.02387 |
| P_adjust(Cpb-Negative_control) | 0.003929 | 0.02387 |
| P_value(Indoor-Negative_control) | 0.03316 | 0.5613 |
| P_adjust(Indoor-Negative_control) | 0.09948 | 0.5613 |
| P_value(Negative_control-Po) | 0.01692 | 0.5613 |
| P_adjust(Negative_control-Po) | 0.03385 | 0.6735 |
| P_value(Soil-Wo) | 0.0009069 | 0.04057 |
| P_adjust(Soil-Wo) | 0.001409 | 0.04868 |
| P_value(Cpb-Wo) | 0.0009229 | 0.0009391 |
| P_adjust(Cpb-Wo) | 0.001127 | 0.001127 |
| P_value(Indoor-Wo) | 0.02395 | 1 |
| P_adjust(Indoor-Wo) | 0.1437 | 1 |
| P_value(Po-Wo) | 0.4309 | 0.7929 |
| P_adjust(Po-Wo) | 0.7929 | 0.7929 |
| P_value(Cpb-Soil) | 0.000891 | 0.01008 |
| P_adjust(Cpb-Soil) | 0.001396 | 0.0121 |
| P_value(Indoor-Soil) | 0.0009069 | 0.4948 |
| P_adjust(Indoor-Soil) | 0.001409 | 0.5635 |
| P_value(Po-Soil) | 0.0009069 | 0.1893 |
| P_adjust(Po-Soil) | 0.001409 | 0.2271 |
| P_value(Cpb-Indoor) | 0.0009229 | 0.007406 |
| P_adjust(Cpb-Indoor) | 0.001396 | 0.008887 |
| P_value(Cpb-Po) | 0.0009229 | 0.002762 |
| P_adjust(Cpb-Po) | 0.001396 | 0.003314 |
| P_value(Indoor-Po) | 0.5635 | 0.7929 |
| P_adjust(Indoor-Po) | 0.7638 | 0.7929 |
